# 3’-hydroxy-4’-methoxy-β-methyl-β-nitrostyrene inhibits tumorigenesis in colorectal cancer cells through ROS-mediated DNA damage and mitochondrial dysfunction

**DOI:** 10.18632/oncotarget.14996

**Published:** 2017-02-02

**Authors:** Chun-Hao Tsai, Amos C. Hung, Yuan-Yin Chen, Ya-Wen Chiu, Pei-Wen Hsieh, Yi-Chen Lee, Yu-Han Su, Po-Chih Chang, Hu Stephen Chu-Sung, Shyng-Shiou F. Yuan

**Affiliations:** ^1^ Translational Research Center, Kaohsiung Medical University Hospital, Kaohsiung Medical University, Kaohsiung, Taiwan; ^2^ Graduate Institute of Medicine, College of Medicine, Kaohsiung Medical University, Kaohsiung, Taiwan; ^3^ Graduate Institute of Natural Products, School of Traditional Chinese Medicine, and Graduate Institute of Biomedical Sciences, College of Medicine, Chang Gung University, Taoyuan, Taiwan; ^4^ Department of Anatomy, School of Medicine, College of Medicine, Kaohsiung Medical University, Kaohsiung, Taiwan; ^5^ Division of General Surgery, Department of Surgery, E-Da Hospital/I-Shou University, Kaohsiung, Taiwan; ^6^ Department of Dermatology, Kaohsiung Medical University Hospital, Kaohsiung Medical University, Kaohsiung, Taiwan; ^7^ Department of Dermatology, College of Medicine, Kaohsiung Medical University, Kaohsiung, Taiwan; ^8^ Department of Obstetrics and Gynecology, Kaohsiung Medical University Hospital, Kaohsiung Medical University, Kaohsiung, Taiwan

**Keywords:** β-nitrostyrene, cell cycle, ROS, colorectal cancer, DNA damage

## Abstract

The β-nitrostyrene family has been shown to suppress cell proliferation and induce apoptosis in types of various cancers. However, the mechanisms underlying the anticancer effects of β-nitrostyrenes in colorectal cancer remain poorly understood. In this study, we synthesized a β-nitrostyrene derivative, CYT-Rx20 (3’-hydroxy-4’-methoxy-β-methyl-β-nitrostyrene), and investigated its anticancer activities in human colorectal cancer cells both *in vitro* and *in vivo*. Our findings showed that treatment with CYT-Rx20 reduced cell viability and induced DNA damage in colorectal cancer cells. In addition, CYT-Rx20 induced cell cycle arrest of colorectal cancer cells at the G2/M phase and upregulated the protein expression of phospho-ERK, cyclin B1, phospho-cdc2 (Tyr15), aurora A, and aurora B, while it downregulated the expression of cdc25A and cdc25C. Furthermore, we found that CYT-Rx20 caused accumulation of intracellular reactive oxygen species (ROS) and reduction of mitochondrial membrane potential. The effects of CYT-Rx20 on cell viability, DNA damage, and mitochondrial membrane potential were reversed by pretreatment with the thiol antioxidant *N*-acetyl-L-cysteine (NAC), suggesting that ROS-mediated DNA damage and mitochondrial dysregulation play a critical role in these events. Finally, the nude mice xenograft study showed that CYT-Rx20 significantly reduced tumor growth of implanted colorectal cancer cells accompanied by elevated protein expression of aurora A, aurora B, γH2AX, phosphor-ERK, and MDA in the tumor tissues. Taken together, these results suggest that CYT-Rx20 may potentially be developed as a novel β-nitrostyrene-based anticancer agent for colorectal cancer.

## INTRODUCTION

Colorectal cancer is one of the most common cancers worldwide with a five-year survival rate of less than 65% [1]. Surgical resection provides curative treatment for early colorectal cancer, while chemotherapeutic agents including platinum-based oxaliplatin and non-platinum-based capecitabine or 5-fluorouracil are commonly used for the treatment of stage III to IV patients [2–4]. In addition, targeted anti-angiogenic agents, such as bevacizumab (Avastin), have been used for the treatment of colorectal cancer [5]. However, chemoresistance remains a major obstacle in the management of advanced colorectal cancer, and therefore further development of effective and novel chemotherapeutic agents is required [6].

The β-nitrostyrene family and its derivatives have been found to exert various biological effects including antimicrobial, antiplatelet, anti-inflammatory, and anticancer activities [7–13]. For example, 3, 4-methylenedioxy-β-nitrostyrene exhibited inhibitory effects on ATPase and decreased inflammasome activation [10]. Regarding anticancer activities, 3,4-methylenedioxy-β-nitrostyrene inhibited β1 integrin and surface protein disulfide isomerase, resulting in suppression of breast cancer cell adhesion and migration [7]. In addition, NTS1 and NTS2, two β-nitrostyrene derivatives, induced cytochrome c release from the mitochondria and promoted apoptosis of Ehrlich ascitic tumor cells [8]. Furthermore, a series of 2-aryl-3-nitro-2*H*-chromenes synthesized as hybrid analogs of β-nitrostyrene and flavanone caused cytotoxicity in breast cancer cells by induction of DNA damage and caspase-3 activity [12].

In our previous study, we reported that CYT-Rx20, a synthetic derivative of β-nitrostyrene, showed inhibitory effect on platelet aggregation and cytotoxicity against breast cancer cells [13, 14]. In this study, we explored the effects of CYT-Rx20 on colorectal cancer and its underlying mechanisms both *in vitro* and *in vivo*.

## RESULTS

### CYT-Rx20 induced cytotoxicity and DNA damage in human colorectal cancer cells

**Figure 1:**
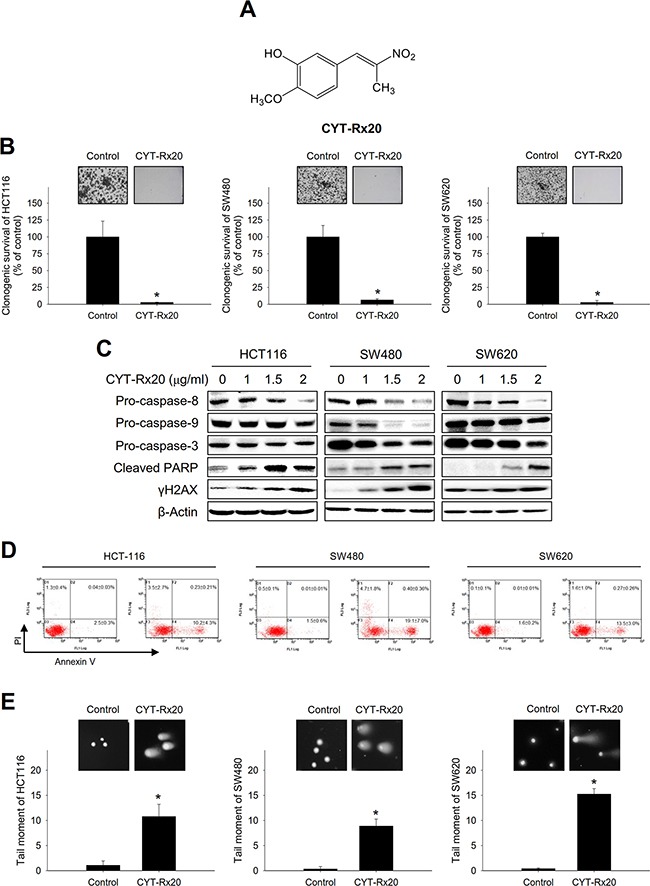
Effects of CYT-Rx20 on cell viability and DNA damage in colorectal cancer cells **A**. Chemical structure of CYT-Rx20. **B**. Cells were treated with CYT-Rx20 (0 or 1.5 μg/ml) for 24 h and allowed to grow for 10 days before crystal violet staining for quantification of clonogenicity. **C**. Caspase-associated proteins were analyzed by immunoblotting after cells were treated with the indicated concentrations of CYT-Rx20 for 24 h. **D**. Cells were treated with CYT-Rx20 (0 or 2 μg/ml) for 24 h, and cell death was examined by Annexin V/PI staining followed by flow cytometric analysis. **E**. DNA double-strand breaks were determined by neutral comet assay after cells were treated with CYT-Rx20 (0 or 1.5 μg/ml) for 24 h. The data were presented as mean±SD. The images were minimally processed (e.g. Brightness and contrast) and applied equally across the entire image. *, significant difference (*p* < 0.05) compared with the control group by Student's t test.

Five β-nitrostyrene derivatives (Figure 1A and Supplementary Figure 1A) were synthesized according to our previous report [13], and their cytotoxic effects on human colorectal cancer cells were analyzed by XTT assay (Supplementary Table 1). Among these compounds, CYT-Rx20 (Figure 1A) exhibited the most potent cytotoxic effect against colorectal cancer cells (Supplementary Table 1). The half inhibition concentrations of CYT-Rx20 in three colorectal cancer cell lines, HCT116, SW480, and SW620, were 1.15±0.15, 1.57 ± 0.06 and 1.51 ± 0.02 μg/mL, respectively (Table 1). In addition, CYT-Rx20 exhibited higher potency against colorectal cancer cells than the clinical chemotherapeutic agents cisplatin and 5-fluorouracil (5-FU) (Table 1). Colony formation assay also showed a significant inhibition of clonogenicity in colorectal cancer cells after CYT-Rx20 treatment (Figure 1B). Moreover, lower cytotoxicity was observed in differentiated Caco-2 cells as compared to its undifferentiated counterparts (Supplementary Table 2).

**Table 1:** Cytotoxicity^a^ of CYT-Rx20, cisplatin, and 5-fluorouracil on human colorectal cancer cell lines

	IC50^b^ in μg/ml (μM)		
	HCT116	SW480	SW620
CYT-Rx20	1.15±0.15 (5.49±0.71)	1.57±0.06 (7.50±0.28)	1.51±0.02 (7.21±0.09)
Cisplatin	6.26±0.14 (20.86±0.46)	6.34±0.60 (21.12±1.99)	9.21±0.54 (30.69±1.79)
5-Fluorouracil	3.04±0.32 (23.37±2.46)	2.96±0.15 (22.75±1.15)	5.18±0.49 (39.82±3.76)

^a^ Cells were treated with various concentrations of CYT-Rx20, cisplatin, or 5-fluorouracil for 24 h before assessment with XTT assay.

^b^ Data were presented as mean±SD from three independent experiments.

The occurrence of apoptotic cell death following CYT-Rx20 treatment was evaluated. Our results showed that CYT-Rx20 decreased the protein expression of pro-caspase-8, −9, and −3 and increased the protein expression of cleaved caspase-8, −9, and −3 (Figure 1C and Supplementary Figure 1B), and pretreatment with the pan-caspase inhibitor Q-VD-OPh attenuated CYT-Rx20-induced cytotoxicity (Supplementary Figure 1C). Moreover, CYT-Rx20 induced cell apoptosis was shown by Annexin V/PI staining (Figure 1D).

We also observed that CYT-Rx20 increased the expression of cleaved PARP and γH2AX, two DNA damage-responding proteins (Figure 1B). The DNA damage caused by CYT-Rx20 was further examined by neutral comet assay (Figure 1E), which showed that treatment with CYT-Rx20 resulted in DNA double strand breaks in colorectal cancer cells.

### Effect of CYT-Rx20 on cell cycle progression and G2/M regulatory proteins

**Figure 2:**
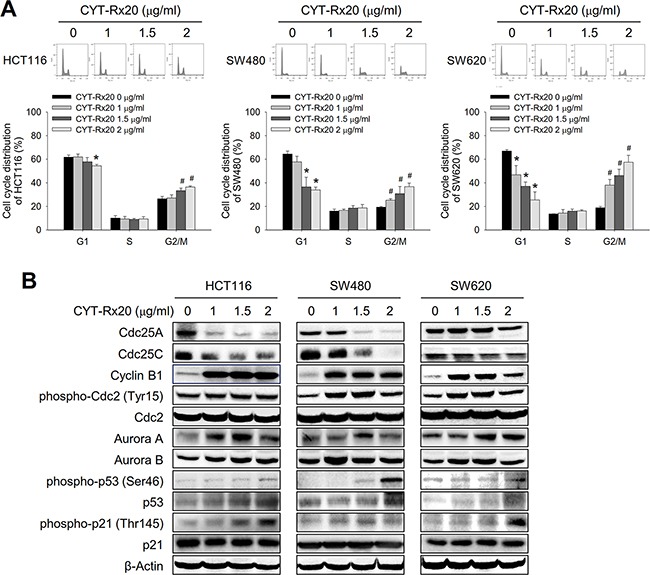
Effects of CYT-Rx20 on cell cycle distribution and regulatory proteins in colorectal cancer cells **A**. Cells were treated with the indicated concentrations of CYT-Rx20 for 24 h, and the cell cycle distribution was examined with propidium iodide staining followed by flow cytometric analysis. **B**. Cell cycle regulatory proteins were analyzed by immunoblotting after cells were treated with the indicated concentrations of CYT-Rx20 for 24 h. The data were presented as mean±SD. The images were minimally processed (e.g. Brightness and contrast) and applied equally across the entire image. * and ^#^, significant difference (*p* < 0.05) compared with the corresponding control group in G1 and G2/M phases, respectively, by Student's t test.

To explore the mechanisms underlying the cytotoxic effects of CYT-Rx20, we next examined the cell cycle distribution profile of CYT-Rx20-treated colorectal cancer cells. The results revealed that CYT-Rx20 treatment led to a significant accumulation of cells at the G2/M phase in a dose-dependent manner (Figure 2A). In agreement with this finding, the expression of cyclin B1, phospho-cdc2 (Tyr15), aurora A, aurora B, phospho-p53 (Ser46), and phospho-p21 (Thr145) were significantly increased, while the expression of cdc25A and cdc25C were decreased after CYT-Rx20 treatment (Figure 2B).

### Involvement of ROS-mediated pathways in CYT-Rx20-induced cytotoxicity

**Figure 3:**
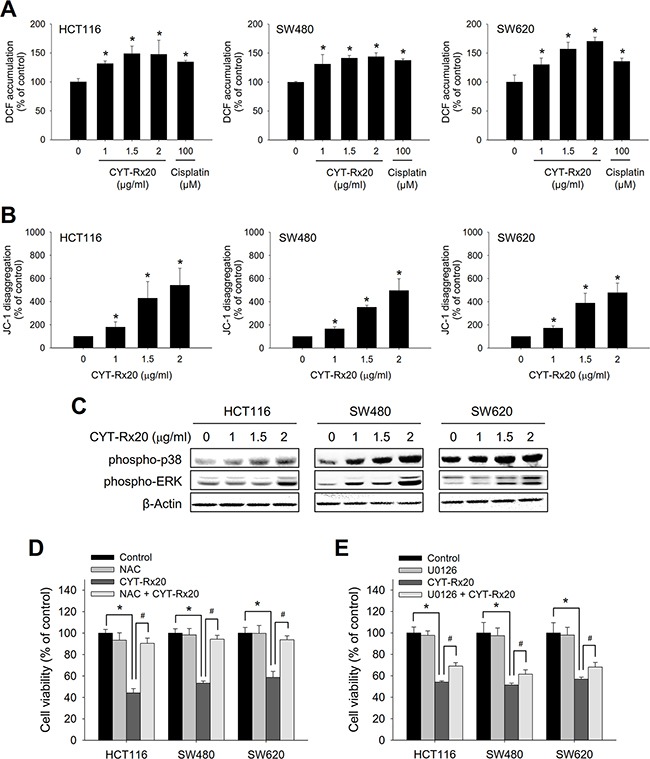
Involvement of ROS accumulation, mitochondrial dysfunction, and MEK/ERK activation in CYT-Rx20-induced cytotoxicity in colorectal cancer cells **A**. The level of ROS was measured by labeling cells with H_2_DCFDA and analyzed by flow cytometry, after cells were treated with the indicated concentrations of CYT-Rx20 or cisplatin (100 μM) as positive control for 2 h. **B**. JC-1 disaggregation representing the loss of mitochondrial membrane potential was measured after cells were treated with the indicated concentrations of CYT-Rx20 for 24 h. **C**. Cells were treated with the indicated concentrations of CYT-Rx20 for 24 h, followed by immunoblotting analysis for the expression of phospho-p38 and phospho-ERK. **D**. Cells were pretreated with NAC (10 mM) for 1 h, followed by CYT-Rx20 (1.5 μg/mL) treatment for 24 h prior to XTT assay. **E**. Cells were pretreated with U0126 (10 μM) for 1 h, followed by CYT-Rx20 (1.5 μg/mL) treatment for 24 h prior to XTT assay. The data were presented as mean±SD. The images were minimally processed (e.g. Brightness and contrast) and applied equally across the entire image. * and ^#^, significant difference (*p* < 0.05) compared with the indicated group by Student's t test.

Mitochondrial dysfunction may trigger MAPK signaling pathways through elevated intracellular reactive oxygen species (ROS) [15, 16]. As shown in Figure 3A, the levels of ROS, observed by labeling cells with H_2_DCFDA, were increased after CYT-Rx20 treatment, accompanied by loss of mitochondrial membrane potential as determined by JC-1 disaggregation (Figure 3B), and increased protein expression of phospho-p38 and phospho-ERK (Figure 3C). The CYT-Rx20-induced mitochondrial dysfunction was further confirmed by increased cytochrome c release (Supplementary Figure 2).

Pretreatment with NAC, a thiol antioxidant, markedly blocked the CYT-Rx20-induced cytotoxicity (Figure 3D), and pretreatment with MEK/ERK inhibitor U0126 partially suppressed CYT-Rx20-induced cell death (Figure 3E). However, pretreatment with p38 inhibitor SB203580 showed no significant effects (Supplementary Figure 1D). As NAC is a thiol antioxidant, we carried out further cytotoxicity experiments with the use of glutathione (GSH) and 2-mercaptoethanol (2-ME), two other thiol antioxidants [17, 18], and the results showed that CYT-Rx20-induced cytotoxicity was largely reversed in the presence of either GSH or 2-ME (Supplementary Figure 1E).

**Figure 4:**
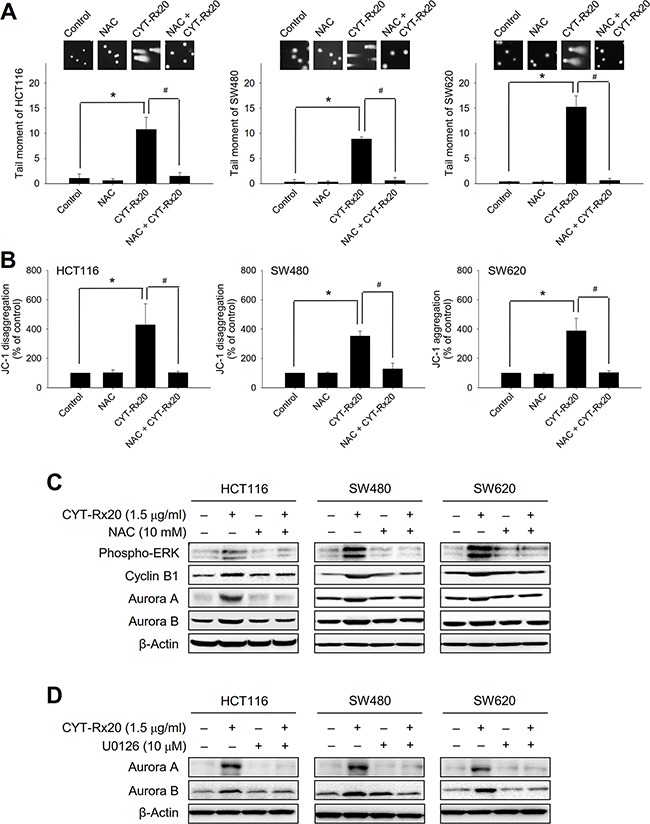
Effects of NAC and U0126 on CYT-Rx20-induced DNA damage, loss of mitochondrial membrane potential, and expression of cell cycle regulatory proteins **A**. Cells were pretreated with NAC (10 mM) for 1 h, followed by CYT-Rx20 (1.5 μg/mL) treatment for 24 h prior to examination of DNA damage by neutral comet assay. **B**. Cells were pretreated with NAC (10 mM) for 1 h, followed by CYT-Rx20 (1.5 μg/mL) treatment for 24 h prior to JC-1 staining and flow cytometric analysis. **C**. Cells were pretreated with NAC (10 mM), followed by CYT-Rx20 (1.5 μg/mL) treatment for 24 h prior to immunoblotting analysis for the expression of phospho-ERK, cyclin B1, aurora A, and aurora B. **D**. Cells were pretreated with U0126 (10 μM), followed by CYT-Rx20 (1.5 μg/mL) treatment for 24 h prior to immunoblotting analysis for the expression of aurora A and aurora B. The images were minimally processed (e.g. Brightness and contrast) and applied equally across the entire image. * and ^#^, significant difference (*p* < 0.05) compared with the indicated group by Student's t test.

We further determined the involvement of ROS in CYT-Rx20-induced DNA damage, mitochondrial membrane potential loss, and protein expression of associated signaling molecules. The results showed that NAC significantly suppressed DNA double strand breaks (Figure 4A) and loss of mitochondrial membrane potential (Figure 4B) caused by CYT-Rx20 treatment. In addition, NAC reversed the effects of CYT-Rx20 on protein expression of phospho-ERK, cyclin B1, aurora A, and aurora B (Figure 4C). Moreover, pretreatment with MEK/ERK inhibitor U0126 blocked the CYT-Rx20-induced increases in aurora A and aurora B expression (Figure 4D). These results suggested that the anticancer effects of CYT-Rx20 on colorectal cancer cells were mediated through ROS-dependent DNA damage and mitochondrial dysfunction with the involvement of MEK/ERK signaling pathway.

### CYT-Rx20 inhibited *in vitro* anchorage-independent cell growth and *in vivo* tumor growth

**Figure 5:**
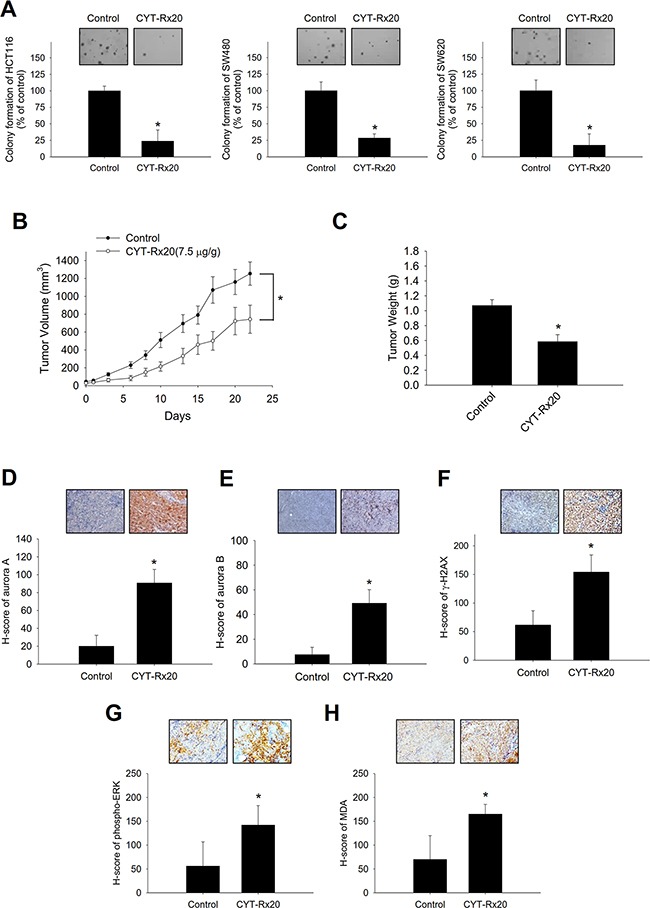
Effects of CYT-Rx20 on *in vitro* anchorage-independent cell growth and *in vivo* xenograft tumor growth in HCT116 colorectal cancer cells **A**. Cells were treated with CYT-Rx20 (1.5 μg/mL) for 24 h, followed by evaluation of anchorage-independent colony formation using soft agar assay. **B**. Female nude mice subcutaneously xenografted with HCT116 cells were intraperitoneally treated with normal saline (control) or 7.5 μg/g of CYT-Rx20 three times per week (n = 10 for each group). Tumor volume was measured every two to three days for each group and calculated according to the formula of width^2^×length/2. **C**. Tumor weight was measured after sacrifice of the mice at the end of the 22-day treatment period. **D** – **H**. Xenograft tumor tissues were analyzed for the expression of aurora A (D), aurora B (E), γH2AX (F), phosphor-ERK (G), and MDA (H) by immunohistochemistry. H-score was calculated as the product of percentage of stained cells and intensity of staining. Bars represent 100 μm. The images were minimally processed (e.g. Brightness and contrast) and applied equally across the entire image. *, significant difference (*p* < 0.05) compared with the control group by Student's t test.

The results of soft agar assay revealed that CYT-Rx20 suppressed the anchorage-independent growth of colorectal cancer cells (Figure 5A). To further investigate the anticancer activity of CYT-Rx20 *in vivo*, the nude mice model with subcutaneous xenograft was employed. As shown in Figure 5B, HCT116 tumor growth was significantly inhibited by CYT-Rx20 treatment in comparison with the control group. After 22 days of CYT-Rx20 treatment, the average tumor volumes for the control and CYT-Rx20 (7.5μg/g) groups were 1254.25±129.84 and 721.60±166.88 mm^3^ (Figure 5B), respectively, and the average tumor weights were 1.07±0.08 and 0.53±0.13 g, respectively (Figure 5C). Furthermore, the expression levels of aurora A (Figure 5D), aurora B (Figure 5E), γH2AX (Figure 5F), phospho-ERK (Figure 5G), and ROS marker malondialdehyde (MDA; Figure 5H) in xenograft tumor sections were increased after CYT-Rx20 treatment. The mice treated with CYT-Rx20 did not show significant changes in body weight (Supplementary Figure 3A), histology of colon, liver, spleen, kidney, heart, and lung (Supplementary Figure 3B), or hematological and biochemical profiles (Supplementary Table 3) compared with the control mice. Furthermore, the expression of cleaved caspase-3 in colon tissue of CYT-Rx20-treated mice was similar to that of untreated mice (Supplementary Figure 3C).

## DISCUSSION

Intracellular ROS has multi-faceted functions both physiologically and pathologically, and physiological levels of ROS are associated with cellular redox reactions and energy metabolism for cell survival [19, 20]. However, in the presence of environmental stress, ROS can be overproduced and may interfere with the stability of mitochondrial membrane potential, leading to an excessive release of mitochondrial ROS which further promotes cell death [19, 21–23]. Therefore, the development of chemotherapeutics that elicit intracellular ROS accumulation may represent a potential strategy for inhibition of tumor growth [24]. Although a variety of compounds have been found to target colorectal cancer cells via ROS production [25–27], information is not available regarding the role of ROS in the anticancer activities of β-nitrostyrenes. In this study, we provided evidence that ROS was critically involved in the anticancer effects of CYT-Rx20 on colorectal cancer cells. GSH is the most abundant non-protein thiol in cells and plays a major role in oxidative stress and redox metabolism [28]. Previous studies indicated that redox metabolism is critical for cancer cells, and modulation of GSH and/or GST isozymes is an ongoing therapeutic strategy in cancer chemotherapy [28]. We also found that the CYT-Rx20-induced cytotoxicity was significantly rescued by thiol antioxidants such as NAC, glutathione, and 2-mercaptoethanol, suggesting that the anticancer activities of CYT-Rx20 may result from the imbalance of thiol redox status [28].

The caspase family mediates apoptotic programmed cell death in colorectal and other cancers, and has been proposed as a therapeutic target for cancer treatment [29–31]. Previous reports indicated that β-nitrostyrene derivatives exhibited anticancer activities via induction of apoptosis [8, 12, 32]. In agreement with these reports, our current data revealed that colorectal cancer cells treated with CYT-RX20 underwent caspase-associated apoptotic cell death. In addition, the CYT-Rx20-treated colorectal cancer cells increased DNA damage and mitochondrial dysfunction, and both were mediated through an ROS-dependent manner, further confirming the important involvement of ROS in these CYT-Rx20-induced cytotoxic events.

Aurora A and aurora B, two critical regulators for mitotic spindle formation, were found to be abnormally increased during G2/M arrest [33–35]. Our results showed that CYT-Rx20 induced G2/M arrest in colorectal cancer cells with upregulated expression of cyclin B1, aurora A, and aurora B, and downregulated expression of cdc25A and cdc25C, which collectively could contribute to the inactivation of cdc2. Tumor suppressor gene p53 and its downstream effector p21 were also activated after CYT-Rx20 treatment. These results provided mechanistic explanation for the antiproliferative effects of CYT-Rx20 on colorectal cancer cells. Activation of ERK is essential for cell cycle progression under normal circumstances [36, 37]. However, over-activation of ERK may result in suppression of cell cycle progression by alteration of a complex network involving various transcription factors and cell cycle regulators [36]. Our current data revealed that CYT-Rx20-induced ERK phosphorylation as well as aurora A and aurora B expression were inhibited by NAC and MEK/ERK inhibitor U0126 in colorectal cancer cells. These data suggested that ROS/MEK/ERK signaling may mediate the anti-mitotic effect of CYT-Rx20 on colorectal cancer cells. It will be valuable undertake further investigations into the unidentified ERK-regulated molecules that participate in the course of cell cycle arrest by CYT-Rx20.

We noticed that the levels of phospho-ERK and phospho-p38 were both increased after exposure to CYT-Rx20. Nevertheless, pretreatment with MEK/ERK inhibitor U0126, but not p38 inhibitor SB203580, suppressed CYT-Rx20-induced cytotoxicity. The results suggested that p38 activation may be triggered indirectly at a late phase of cell death following CYT-Rx20 treatment in colorectal cancer cells, and therefore blocking p38 signaling did not reverse CYT-Rx20-induced cytotoxicity.

Finally, our results revealed that CYT-Rx20 suppressed *in vivo* tumor growth of the xenograft colorectal cancer cells without obvious impairment of hematopoiesis and renal or liver functions, nor did it cause obvious histological changes in colon or major organs of nude mice, rendering CYT-Rx20 a potentially low toxic anti-colorectal cancer agent.

## CONCLUSIONS

The present study demonstrated that the synthetic β-nitrostyrene derivative CYT-Rx20 impaired cell cycle progression and inhibited colorectal cancer cell growth through a ROS-mediated pathway involving DNA damage and mitochondrial dysfunction. Future pre-clinical studies are required to confirm the usefulness of CYT-Rx20 as a potential β-nitrostyrene-based chemotherapeutic agent for human colorectal cancer.

## MATERIALS AND METHODS

### Reagents

CYT-Rx20 was synthesized according to our previous report [13]. Dulbecco's Modified Eagle medium (DMEM), H_2_DCFDA and JC-1 were purchased from Invitrogen (Carlsbad, CA, USA). Fetal bovine serum, penicillin, streptomycin, and amphotericin B were purchased from Biological Industries (Beit Haemek, Israel). XTT, propidium iodide, *N*-acetyl-L-cysteine (NAC), glutathione (GSH), 2-mercaptoethanol (2-ME), U0126, DMSO, and Q-VD-OPh were purchased from Sigma-Aldrich (St Louis, MO, USA). Other reagents employed in this study were indicated separately wherever suitable.

### Cell culture

Human colorectal cancer cell lines, HCT116, SW480, and SW620, were obtained from Bioresource Collection and Research Center of Taiwan, and cultured in DMEM medium supplemented with 10% fetal bovine serum, 100 U/mL penicillin, 100 μg/mL streptomycin, and 2.5 μg/mL amphotericin B at 37°C in a 5% CO_2_ incubator [38].

### Caco-2 differentiation

Human Caco-2 was maintained at low-density as previously described [38]. For differentiation assay, cells were seeded on polycarbonate filters with 0.4 μm pore diameter (Transwell, Corning Inc. Lowell, MA, USA) at 3.5 × 10^5^ cells/cm^2^ for 21 days.

### XTT cell viability assay

Cells were seeded at 6×10^3^ cells/well in 96-well plates and allowed to attach overnight. After treatment with CYT-Rx20, cell viability was measured by the XTT assay according to our previous report [39].

### Colony formation assay

Cells were seeded at a density of 500 cells/well in 96-well plates, followed by treatment with CYT-Rx20 for 24 h. After rinsing with fresh medium, cells were allowed to grow for 10 days and then stained with 0.5% crystal violet. Cell culture medium was refreshed every 2-3 days during the incubation period.

### Immunoblotting analysis

The procedures for immunoblotting analysis were described in a previous report [40]. Antibodies against aurora A, aurora B, phospho-cdc2 (Tyr15), p21, phospho-p21 (Thr145), p53, phospho-p53 (Ser46), γH2AX (Ser139), cyclin B1, and phospho-ERK1/2 (Thr202/Tyr204) were purchased from GeneTex (Irvine, CA, USA). Antibodies against cleaved caspase-3, cleaved caspase-8, cleaved caspase-9, caspase-9, cleaved PARP, cdc25A, cdc25C, and phospho-p38 (Thr180/Tyr182) were purchased from Cell Signaling (Danvers, MA, USA). Antibodies against caspase-8 and caspase-3 were purchased from Novus Biologicals (Littleton, CO, USA).

### Neutral comet assay for detection of DNA double-strand breaks (DSBs)

DSBs were determined by neutral comet single-cell gel electrophoresis according to a previous report [41]. Cells treated with CYT-Rx20 for 24 h were combined with 1% low melting point agarose at a ratio of 1:10 (v/v), and 75 μl of the mixture was immediately pipetted onto CometSlide (Trevigen; Gaithersburg, MD, USA) at 4°C and placed in the dark for 10 min. The slides were then immersed in ice-cold lysis solution (Trevigen) for 30 min in 50 mL of 1X TBE (90 mM Tris-HCl, 90 mM boric acid, and 2 mM EDTA, pH 8.0). Finally, slides were transferred from 1X TBE buffer, placed in a horizontal electrophoresis apparatus at 20 V for 10 min, and stained with 1:10,000 DAPI (Sigma-Aldrich). The averaged tail moment was analyzed by CometScore software (TriTek; Sumerduck, VA, USA).

### Cell cycle analysis

Cells treated with the indicated concentrations of CYT-Rx20 for 24 h were harvested, fixed, and stained with propidium iodide as described in a previous report [42], followed by flow cytometric analysis (FC 500 MCL, Beckman Coulter, Brea, CA, USA).

### Detection of intracellular ROS and mitochondrial membrane potential

The levels of intracellular ROS and mitochondrial membrane potential were examined by flow cytometry, using H_2_DCFDA and JC-1, respectively. The procedures were described in a previous report [39]. Cells treated with CYT-Rx20 for 2 h were loaded with 20 μM H_2_DCFDA and those treated for 24 h were loaded with 2 μM JC-1, followed by incubation at 37°C for 30 min or 15 min (JC-1) in a dark environment. The cells were then analyzed immediately by flow cytometry (FC 500 MCL, Beckman Coulter, Brea, CA, USA).

### Anchorage-independent soft agar assay

Cells were seeded at 1000 cells/well with 0.25% agar in 48-well plates, followed by treatment with the indicated concentrations of CYT-Rx20 for 24 h. Cells were allowed to grow for 30 days and then stained with 0.5% crystal violet. Cell culture medium was refreshed every 2-3 days during the incubation period.

### *In vivo* tumor xenograft study

Six-week-old female immune-deficient BALB/cAnN.Cg-*Foxn1^nu^*/CrlNarl mice (National Laboratory Animal Center of Taiwan) were subcutaneously injected with 3×10^6^ HCT116 cells into both flanks. When the tumors became visible (approximately at an average diameter of 3 mm), the mice were intraperitoneally injected with 7.5 μg/g of CYT-Rx20 dissolved in 100 μL normal saline three times a week. The control mice were injected with normal saline. Tumor volumes were measured and calculated according to the formula of width^2^×length/2. The animal studies were approved by the Institutional Animal Care and Use Committee (IACUC no. 102009) of Kaohsiung Medical University, Taiwan.

### Immunohistochemistry and hematoxylin & eosin staining

Immunohistochemical staining for the expression of aurora A, aurora B, γH2AX, phosphor-ERK, and MDA in xenograft tumor was performed with the fully automated Bond-Max system according to the manufacturer's instructions (Leica Microsystems, Wetzlar, Germany). For quantification, the sections of each immunostaining were scored using the method of histochemical score (H-score), which was calculated as the product of percentage of stained cells and intensity of staining [43]. In addition, the tissues from various mice organs were observed with hematoxylin & eosin stain.

### Statistical analysis

Quantitative data were presented as mean±SD from three independent experiments. Student's t-test was used to determine the significance of difference between two groups. A *P* value less than 0.05 was considered statistically significant.

## SUPPLEMENTARY MATERIALS FIGURES AND TABLES


