# Supplementary material for: 3’-hydroxy-4’-methoxy-β-methyl-β-nitrostyrene inhibits tumorigenesis in colorectal cancer cells through ROS-mediated DNA damage and mitochondrial dysfunction
Source: Oncotarget. 2017 Feb 2;8(11):18106–17. doi: 10.18632/oncotarget.14996 (PMC5392311; doi:10.18632/oncotarget.14996)
Supplement: Supplementary file 1 [file oncotarget-08-18106-s001.pdf]

3′-hydroxy-4′-methoxy-β-methyl-β-nitrostyrene inhibits tumorigenesis in colorectal cancer cells through ROS-mediated DNA damage and mitochondrial dysfunction

SUPPLEMENTARY FIGURES AND TABLES

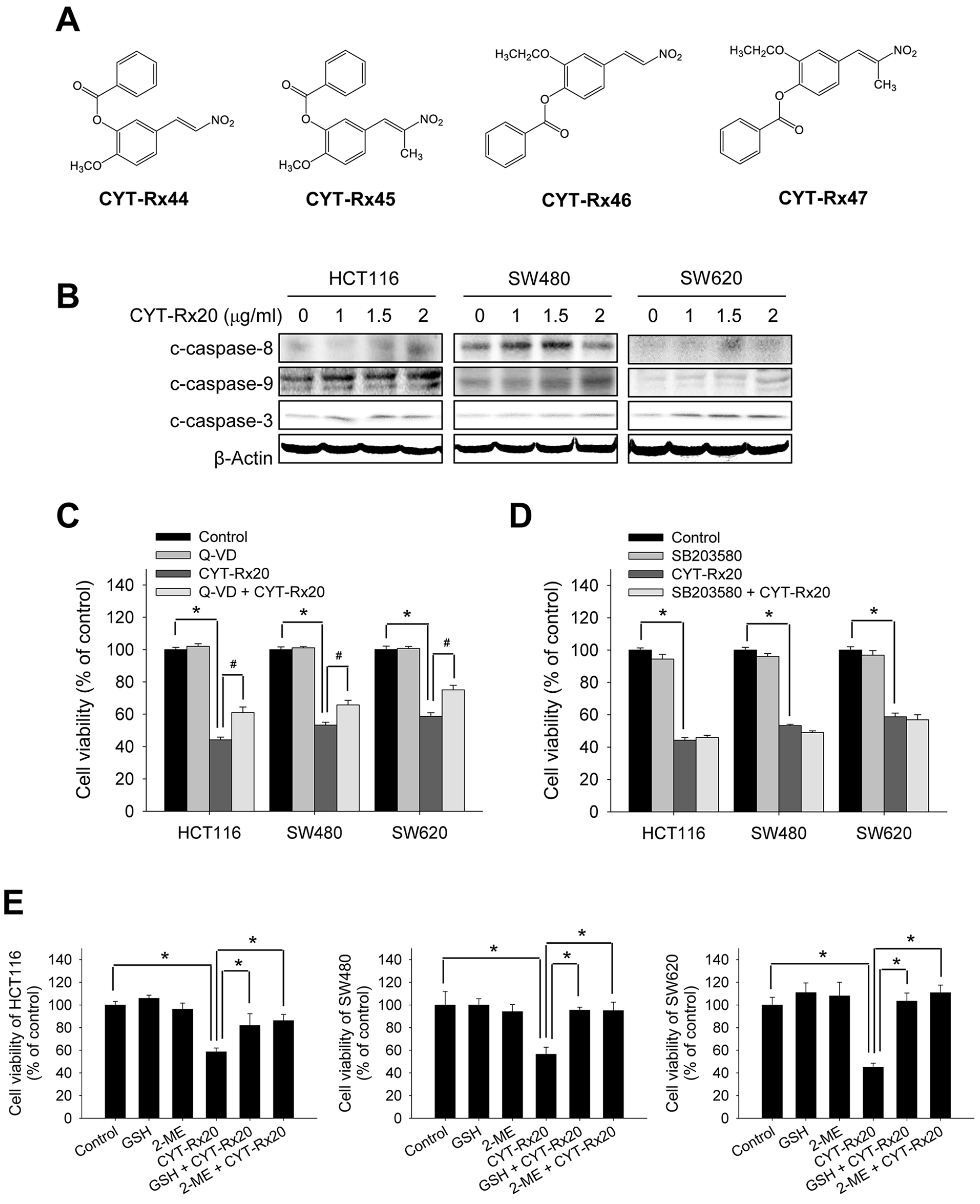

Supplementary Figure 1: A. Chemical structures of CYT-Rx44, CYT-Rx45, CYT-Rx46, and CYT-Rx47. **B.** The expression of cleaved caspases in the colorectal cancer cells were analyzed by immunoblotting after treatment with the indicated concentrations of CYT-Rx20 for 24 h. **C.** Cells were pretreated with Q-VD (10 µM) for 1 h, followed by CYT-Rx20 (1.5 µg/mL) treatment for 24 h prior to XTT assay. **D.** Cells were pretreated with SB203580 (10 µM) for 1 h, followed by CYT-Rx20 (1.5 µg/mL) treatment for 24 h prior to XTT assay. **E.** Cells were pretreated with glutathione (GSH; 1 mM) or 2-mercaptoethanol (2-ME; 100 µM) for 1 h, followed by CYT-Rx20 (1.5 µg/mL) treatment for 24 h prior to XTT assay. The data were presented as mean±SD. * and #, significant difference ($p < 0.05$) compared with the indicated group by Student's t test.

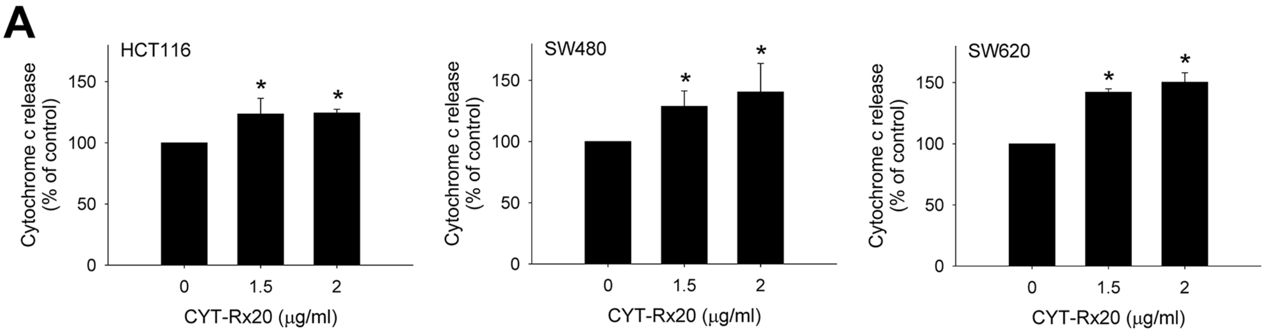

Supplementary Figure 2: Cells were treated with the indicated concentrations of CYT-Rx20 for 24 h. Subsequently, cells were trypsinized and resuspended in PBS, fixation/permeablization with 80% methanol for 10 min, incubated with anti-cytochrome c antibody (Invitrogen; Carlsbad, CA, USA) at 37°C for 30 min and analyzed by flow cytometry. *, significant difference ($p < 0.05$) compared with the control group by Student's t test.

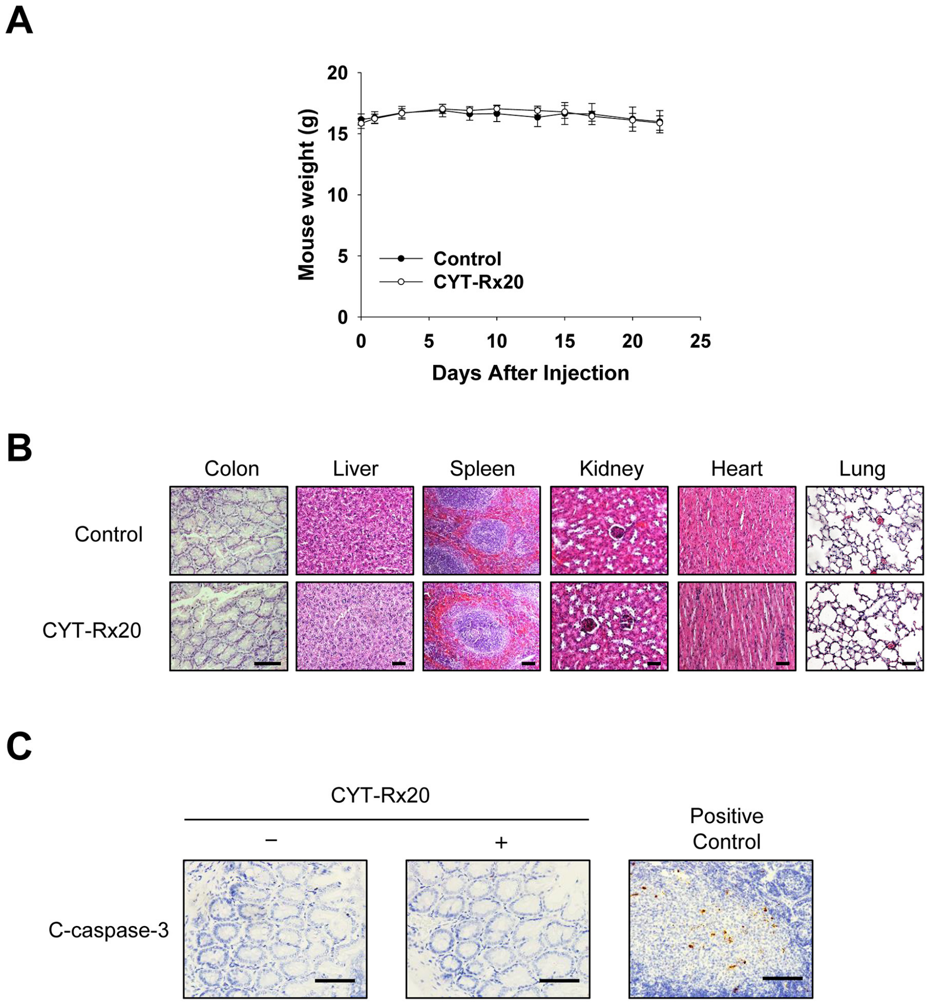

Supplementary Figure 3: A. Six-week-old female immune-deficient BALB/cAnN.Cg-*Foxn1*^{nu}/CrlNarl mice were subcutaneously injected with 3×10^6 HCT116 cells into both flanks, and the body weights were measured. **B.** Hematoxylin and eosin staining of tissues from mice organs. **C.** IHC staining of cleacved-caspase-3 in mouse colorectal tissues with or without CYT-Rx20 treatment. Mouse lymph node stained with cleaved-caspase-3 was used as a positive control. Bars represent 100 μm.

Supplementary Table 1: Cytotoxicity[a] of CYT-Rx20, CYT-Rx44, CYT-Rx45, CYT-Rx46 and CYT-Rx47 on HCT116 human colorectal cancer cell line

	IC_{50}[b] (μg/ml)				
	CYT-Rx20	CYT-Rx44	CYT-Rx45	CYT-Rx46	CYT-Rx47
HCT116	1.15 ± 0.15	>7	3.12 ± 0.12	8.50 ± 1.20	>2.2

[a] Cells were treated with various concentrations of CYT-Rx compounds for 24 h before assessment with XTT assay.
[b] Data was presented as mean ± SD from three independent experiments.

Supplementary Table 2: Cytotoxicity of CYT-Rx20 on Caco-2

	IC_{50}[a] in μg/ml	
	Caco-2	Differentiated Caco-2
CYT-Rx20	2.99 ± 0.03	5.08 ± 0.21

[a]Data were presented as mean ± SD from three independent experiments.

Supplementary Table 3: Blood counts and biochemical profiles[a] of the nude mice after treatment with CYT-Rx20 for 22 days

	Control	CYT-Rx20 (1.5 μg/g)	CYT-Rx20 (7.5 μg/g)
GOT (U/l)	387.3 ± 177.2	648.3 ± 319.4	375.5 ± 88.4
GPT (U/l)	108.3 ± 39.6	216.5 ± 68.6	192.3 ± 71.0
BUN (mg/dl)	36.1 ± 2.5	34.8 ± 2.0	30.2 ± 1.6
Creatinine (mg/dl)	0.5 ± 0.0	0.6 ± 0.1	0.5 ± 0.0
Hb (g/dl)	14.5 ± 0.6	17.0 ± 0.1	15.7 ± 0.3
HCT (%)	43.2 ± 1.6	51.8 ± 0.6	48.6 ± 1.3
MCV (fl)	46.2 ± 0.9	47.3 ± 0.5	49.0 ± 0.9
MCH (pg)	15.5 ± 0.2	15.5 ± 0.1	15.9 ± 0.1
MCHC (g/dl)	33.6 ± 0.4	32.8 ± 0.2	32.4 ± 0.6
WBC (10^3/ul)	9.8 ± 1.8	6.5 ± 0.9	8.9 ± 0.2
RBC (10^6/dl)	9.4 ± 0.3	11.0 ± 0.1	9.9 ± 0.2
PLT (10^3/ul)	613.8 ± 68.6	562.6 ± 21.8	631.0 ± 32.7

[a] Data were presented as mean ± SD.
